# Temperature Interference on ZIKV and CHIKV Cycles in Mosquitoes and Mammalian Cells

**DOI:** 10.3390/pathogens13090814

**Published:** 2024-09-21

**Authors:** Tiago Souza Salles, Erica Santos Martins-Duarte, Marcelo Damião Ferreira de Meneses, Monica Ferreira Moreira, Davis Fernandes Ferreira, Renata Campos Azevedo, Wanderley De Souza, Lucio Ayres Caldas

**Affiliations:** 1 Department of Biochemistry, Institute of Chemistry, Federal University of Rio de Janeiro, Rio de Janeiro 21941-909, Brazil; tiagosouzasalles@gmail.com (T.S.S.); monica@iq.ufrj.br (M.F.M.); 2 Fiocruz Biodiversity and Health Biobank, Oswaldo Cruz Foundation, Rio de Janeiro 21040-361, Brazil; 3 Department of Parasitology, Federal University of Minas Gerais, Belo Horizonte 31270-901, Brazil; emartinsduarte@gmail.com; 4 Department of Virology, Institute of Microbiology, Federal University of Rio de Janeiro, Rio de Janeiro 21941-902, Brazil; marcelomeneses@micro.ufrj.br (M.D.F.d.M.); renatacampos@micro.ufrj.br (R.C.A.); 5 Department of Pathology, Duke University Medical Center, Durham, NC 27710, USA; davis.ferreira@duke.edu; 6 Precision Medicine Research Center, Institute of Biophysics, Federal University of Rio de Janeiro, Rio de Janeiro 21941-904, Brazil; wsouza@biof.ufrj.br; 7 National Centre for Structural Biology and Bioimaging (CENABIO), Federal University of Rio de Janeiro, Rio de Janeiro 21941-902, Brazil; 8 Multidisciplinary Research Centre (Numpex-Bio), Federal University of Rio de Janeiro, Rio de Janeiro 25265-970, Brazil

**Keywords:** Chikungunya virus, Zika virus, arbovírus, virus cycle

## Abstract

Temperature is a determining factor for the viral cycle. In this study, we investigate the effect of different temperatures on the cycles of two important arboviruses—Zika (ZIKV) and Chikungunya (CHIKV)—in Vero (mammalian) and C6/36 (mosquito) cells. We compare genome quantification to infectivity at 28 °C and 37 °C in both cell types. Virus–cell interaction was also examined by transmission electron microscopy, allowing the observation of phenomena such as virus-surfing and giant forms for CHIKV, as well as the the scarcity of ZIKV in C6/36 cells compared to its cycle in mammalian cells.

## 1. Introduction

Despite belonging to different families, the Zika virus (ZIKV, Flavivirus, *Flaviviridae*) and Chikungunya virus (CHIKV, Alphavirus, *Togaviridae*) are arboviruses of significant interest to public health. ZIKV was first discovered in 1947 in a sentinel rhesus monkey in the Zika Forest of Uganda. The virus was subsequently isolated from mosquitoes the following year in 1948 [1]. Notably, the recent outbreak from 2015 to 2016 reached 80 countries, raising concerns about the spread of diseases caused by arboviruses [2].

CHIKV, on the other hand, was isolated in Tanzania and Mozambique in the early 1950s. Encephalitis, myocarditis, meningitis, nephritis, hepatitis, severe arthralgia, and multiple organ failure are some of the most important morbidities observed in humans infected with CHIKV. Despite the low fatality rate, the most common complication of Chikungunya disease—joint pain—can result in decreased quality of life, also affecting the ability to work and the public health dynamics [3].

Despite being initially associated with small outbreaks in Africa, the virus caused epidemics of concern in urban areas of Asia in the 1960s. Later, significant CHIKV epidemics also occurred in Kenya, on the island of La Reunion, and in India, before the first cases were reported in Europe and North and South America [4,5,6,7,8,9]. The three major known lineages of CHIKV are the East, Central, and South African lineage; the West African lineage; and the Asian lineage [10].

Disease caused by ZIKV in humans was, for decades, recognized as a mild febrile or asymptomatic infection until its association with cases of microcephaly, particularly in the northeast region of Brazil. This led to the declaration by the WHO in 2016 of ZIKV as a public health emergency of international concern [11].

CHIKV and ZIKV were previously transmitted mainly by *Ae. aegypti* mosquitoes. However, a single mutation in the gene A226V of the envelope protein E1 led not only to the emergence of the CHIKV Indian Ocean lineage (IOL) but also to CHIKV transmission by *Ae. albopictus*, allowing the spread of this pathogen to other regions of the globe [12]. Although reported more frequently in tropical and subtropical regions, CHIKV is considered a potential risk for reaching temperate regions [13]. Urbanization, human travel, viral adaptation, lack of effective control measures, and the spread of new vectors have contributed to the recent re-emergence of CHIKV infection. An attenuated, single-dose CHIKV IXCHQ vaccine has recently been approved; however, its efficacy is still debated [14].

Although a few studies addressed the consequences of temperature variations on mosquito competence for arbovirus transmission [15,16], the influence of environmental temperatures was also investigated during larval development [17]. While *A. aegypti* is a more competent vector for many arboviruses and is therefore the most studied species, *A. albopictus* is capable of expanding geographically and has a longer lifespan [18].

In this study, we infected Vero and C6/36 cells with ZIKV or CHIKV at 37 °C or 28 °C to investigate the viral RNA production, the release of infectious particles, and the morphogenesis of both arboviruses. These temperatures were chosen because they are the optimal conditions for culturing mammalian and mosquito cells in vitro. By exchanging these conditions, we aimed to create an interesting stress to evaluate the virus cycle in the cell. The times of infection were chosen considering that initial viral entry and early replication stages often occur within the first 24 h, while peak viral loads and replication cycles may be observed around 48 to 72 h post-infection (hpi), taking into account the different timings for these viruses.

## 2. Material and Methods

### 2.1. Cells and Virus

The mammalian Vero cells (African green monkey kidney) and *Ae. albopictus* mosquitoes C6/36 cells were maintained in DMEM (Gibco, Life Technologies; Grand Island, NE, USA) at 37 °C and in Leibovitz L-15 medium (Gibco, Life Technologies; Grand Island, NE, USA) at 28 °C, respectively. Both culture media were supplemented with 10% fetal bovine serum (FBS; Sigma-Aldrich; Burlington, VT, USA), 100 U/mL penicillin, and 100 mg/mL streptomycin.

The CHIKV lineage used in this study was ECSA. The ZIKV lineage used was the African MR766, which differs by approximately 10% at the nucleotide level compared to the Asian lineage [19].

### 2.2. Infection Assays

Semi-confluent (80%) cells were infected with 0.2 MOI (multiplicity of infection) of ZIKV or CHIKV in serum-free medium. After an absorption period of 1.5 h at 37 °C (for Vero cells) or 28 °C (for C6/36 cells) in 5% CO_2_, fresh medium containing 2% FBS was added. At this point, half of the samples were incubated at different temperatures, i.e., infected and mock Vero cells were incubated at 28 °C, while infected and mock C6/36 cells were incubated at 37 °C. At specific hpi, cells were processed for PCR, titration or electron microscopy.

### 2.3. Infectivity Assay

Quantification of infectious viruses in Vero and C6/36 cultures was performed using the tissue culture infectious dose (TCID50) method and calculated according to the Reed-Muench method. TCID50 experiments were conducted in 96-well plates in quadruplicate across three independent experiments.

### 2.4. RT-PCR Assays

C6/36 and Vero cells were infected with CHIKV or ZIKV (MOI = 0.2) for a period of 1 h (adsorption). The inoculum was then removed, and the cells were incubated at different temperatures: C6/36 at 37 °C, C6/36 at 28 °C, Vero at 37 °C, and Vero at 28 °C. After 24, 48, and 72 h, part of the supernatants was removed for viral RNA quantification by RT-qPCR using the DeltaCT method. The RT-PCR test used was a multiplex kit (ZDC kit; Biomanguinhos, Fundação Oswaldo Cruz, Rio de Janeiro, Brazil) designed to detect Zika, Chikungunya, and Dengue in a simultaneous assay. One-way analysis of variance (ANOVA) followed by Tukey’s Multiple Comparison Test (*p* < 0.05) was used in the capture experiments, and Tukey’s test (* = *p* <0.05) was used to compare the differences in RNA amounts at different temperatures. There was no significant variance. Statistical analyses were performed using Prism 8 (GraphPad Software; Boston, MA, USA). All significant values had *p*-values of less than 0.05.

### 2.5. En Bloc Processing for Transmission Electron Microscopy

For transmission electron microscopy (TEM) analysis, mock and infected monolayers in 25 cm^2^ plastic culture flasks were fixed in 2.5% glutaraldehyde in 0.1 M cacodylate buffer (pH 7.2) and post-fixed for 1 h in 1% OsO_4_/0.8% potassium ferrocyanide in the same buffer. Samples were then incubated with 2.5% uranyl acetate in water for 2 h, washed three times in 0.1 M cacodylate buffer (pH 7.2), dehydrated in ethanol, and embedded in Polybed resin (Polysciences; Warrington, PA, USA). Ultrathin sections were stained with 5% uranyl acetate (40 min) and 4% lead citrate (5 min) before observation using an FEI Tecnai T20, an FEI Tecnai G20 or an FEI Tecnai Spirit transmission electron microscope. These microscopes were, respectively, equipped with the following cameras: Megaview 1 K, Eagle 4 K and a Veleta 2 K. Images ranged in magnification from 20,000 to 200,000 times, and pixels sizes varied from 2 to 10 nm.

## 3. Results

To investigate the influence of temperature on the quantity of CHIKV and ZIKV genomes, the supernatants of the infected cells were collected at 24, 48, and 72 hpi, and RT-PCR was performed.

**Figure 1:**
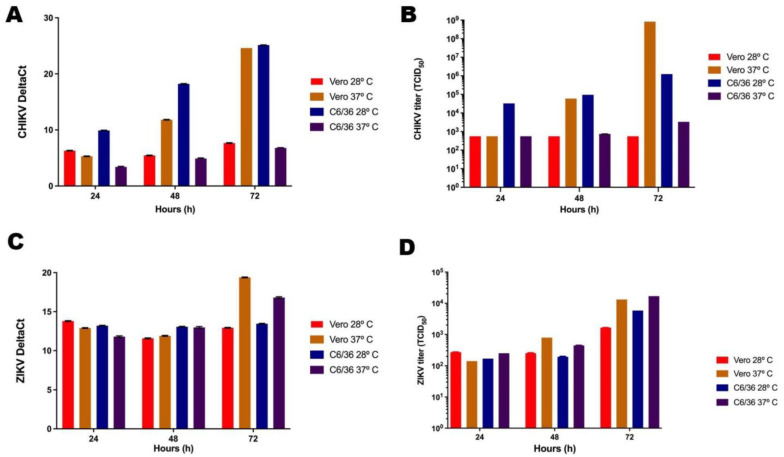
**Quantification of viral RNA and infectious viruses.** RT-PCR was performed to determine the amount of CHIKV (**A**) and ZIKV (**B**) RNA. TCID50 was performed to quantify the number of infectious particles of CHIKV (**C**) and ZIKV (**D**). Due to the small size of the error bars on the logarithmic scale, they are not visible in the graph.

Although at optimal temperatures the supernatants of CHIKV-infected Vero cells revealed lower CHIKV RNA values than those of CHIKV-infected C6/36 cells at 24 and 48 hpi. The values virtually coincide at 72 hpi (Figure 1A). Non-optimal temperatures for Vero and C6/36 cells (28 °C and 37 °C, respectively) resulted in a pronounced decrease in CHIKV RNA concentration. The concentration of viral RNA in C6/36 cells was lower at all three time points (Figure 1A).

For ZIKV, higher RNA concentrations were achieved in Vero cells at 72 hpi and 37 °C. However, this interaction model displayed lower ZIKV RNA concentrations at 24 hpi compared to those obtained from Vero cells at 28 °C and C6/36 cells at 28 °C. Additionally, at 48 hpi, ZIKV RNAs were less abundant in Vero cells at both 37 °C and 28 °C. Only the supernatant of ZIKV-infected C6/36 cells at 37 °C displayed a linear increase in viral RNA concentration over time (Figure 1C).

Although RT-PCR assesses viral RNA, it cannot determine the number of infectious virus particles. Therefore, we quantified infectious viruses using the TCID50 method to evaluate CHIKV and ZIKV production at the temperatures and in the cell lines previously tested for viral RNA concentration.

For CHIKV, the highest overall number of virions was found at a temperature of 37 °C in the supernatant of Vero cells at 72 hpi. However, at 24 and 48 hpi, higher concentrations of infectious CHIKV were present in the supernatant of C6/36 cells at 28 °C. Vero cells infected and incubated at 28 °C exhibited lower concentrations of CHIKV at all time points measured. On the other hand, C6/36 cells at 37 °C produced CHIKV levels similar to those found in Vero cells at 28 °C, except at 72 hpi, when the virus concentration was slightly higher. In Vero cells, no increase in CHIKV titers was observed at 48 and 72 hpi (Figure 1B). The CHIKV RNA load correlated with the results obtained for TCID50; however, it is important to note that in C6/36 cells under standard conditions, the increase in RNA was more pronounced than that observed in the titration of infectious particles (Figure 1A,B).

The optimal temperatures (37 °C for Vero, 28 °C for C6/36) for ZIKV infection in these cell lines displayed a linear increase in viral titers over time, as expected. However, a similar linear increase in ZIKV production was also observed at the non-optimal temperatures (28 °C for Vero, 37 °C for C6/36) (Figure 1D).

**Figure 2:**
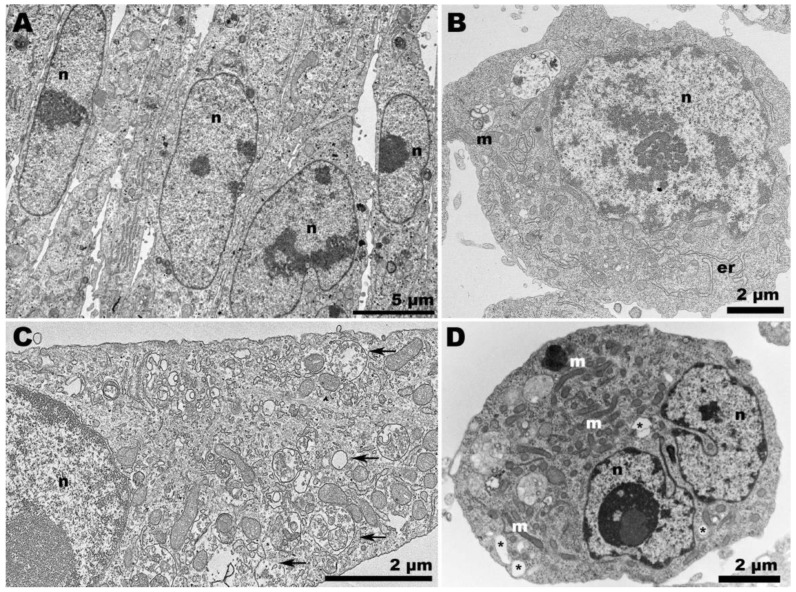
**Transmission electron microscopy of non-infected cells.** (**A**) Vero cells at 37 °C were elongated and adjacent to each other; (**B**) C6/36 cells at 28 °C were rounded and displayed elongated endoplasmic reticulum profiles (er); (**C**) Vero cells at 28 °C exhibited considerable vacuolization (arrows); (**D**) C6/36 cells at 37 °C remained rounded, but vacuolization (asterisk) was also observed, along with an increase in the presence of mitochondria (m). (n): nucleus.

This led us to perform a TEM analysis of the samples to investigate the occurrence of distinct features in the morphogenesis of CHIKV and ZIKV at non-optimal temperature conditions. The distribution of mock cells varied according to the temperature. Vero cells at 37 °C displayed a more clustered arrangement (Figure 2A), compared to those at 28 °C, a condition in which the presence of vacuoles became more noticeable (Figure 2C). C6/36 cells (Figure 2B,D) exhibited considerable vacuolization at 37 °C (Figure 2D).

**Figure 3:**
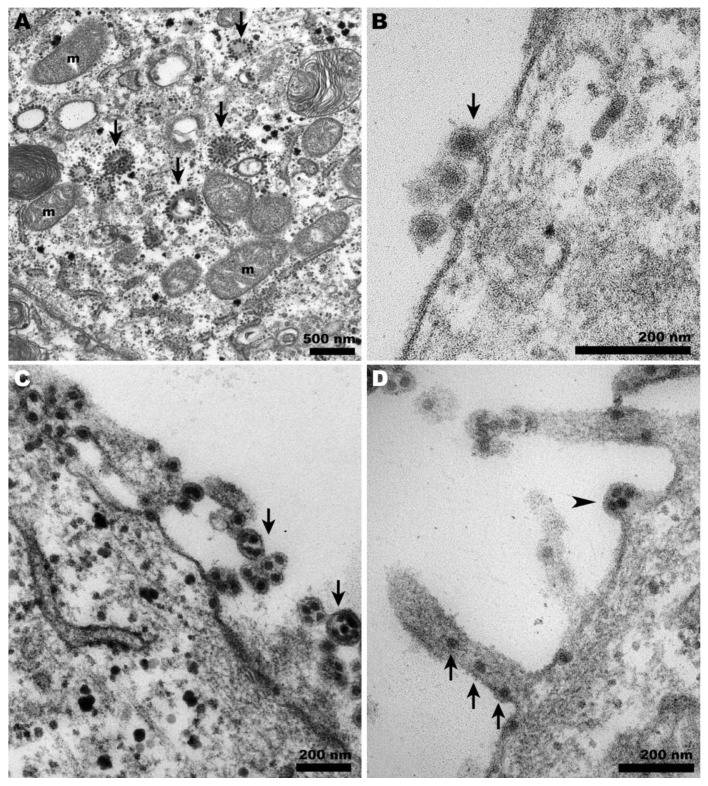
**Transmission electron microscopy of CHIKV-infected Vero cells at 37 °C.** (**A**) CHIKV-induced CPV-II (arrows) surrounded by clusters of mitochondria (m) were abundant in the cytosol; (**B**) characteristic budding of CHIKV at the plasma membrane (arrows); (**C**) giant forms adjacent to the cell surface (arrows); (**D**) budding of giant forms (arrowheads) and cell membrane projections carrying CHIKV (arrows).

At 37 °C, CHIKV-infected Vero cells exhibited the characteristic features of a typical infection in these cells (Figure 3A–C). The presence of CPV-II was abundant (Figure 3A), along with viral budding at the plasma membrane (Figure 3B), the occurrence of giant forms (Figure 3C,D), and virus surfing (Figure 3D).

**Figure 4:**
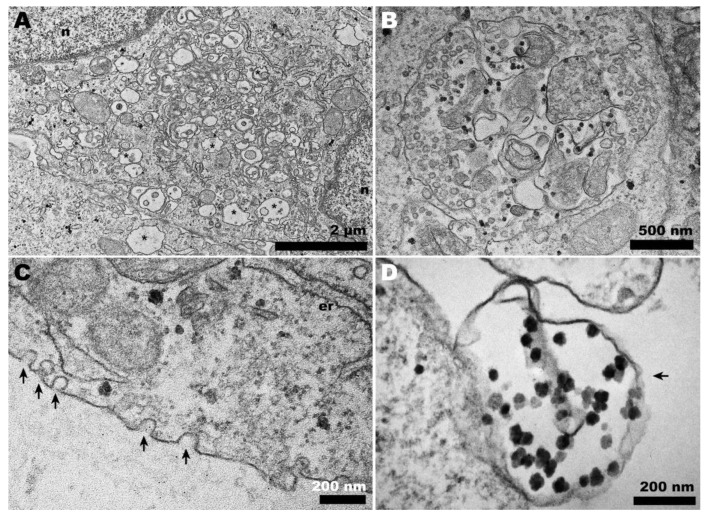
**Transmission electron microscopy of CHIKV-infected Vero cells at 28 °C.** (**A**) Membranous rearrangement observed in the cytoplasm of Vero cells, along with vacuolization (asterisks), leading to the formation of CPV-I (**B**); (**C**) plasma membrane invaginations (arrows) that may give rise to vesicles within CPV-I; (**D**) giant form-like structures, with the outline of a plasma membrane bubble (arrow). (n): nucleus; (er): endoplasmic reticulum.

In contrast, when CHIKV infection occurred in Vero cells at 28 °C, neither CPV-II nor viral budding was observed (Figure 4A–D), even at later time points such as 72 hpi. Spherule-containing vacuoles were frequently present (Figure 4B), along with other features such as endocytic-like structures in the cell plasma membrane (Figure 4C), which seem to precede CPV-II formation. Structures resembling giant forms were associated with the cell surface (Figure 4D), but they contained significantly different contents compared to those observed at the optimal temperature (Figure 3C).

**Figure 5:**
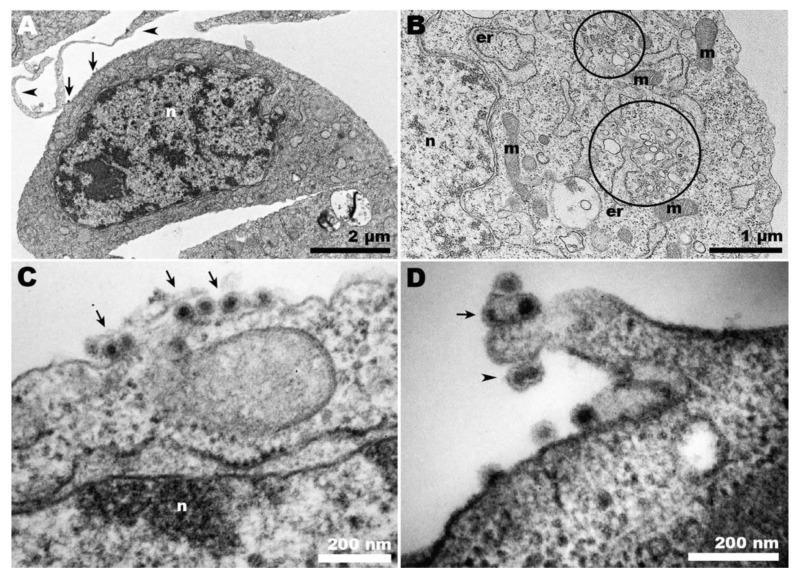
**Transmission electron microscopy of CHIKV-infected C6/36 cells at 28 °C.** (**A**) Panoramic view of infected cells showing condensed chromatin (n), virus adhered to the cell surface (arrows), and cell membrane projections from adjacent cells (arrowheads); (**B**) CHIKV-induced extensive vesiculation (circles); (**C**) CHIKV (arrows) adhered to the cell surface; (**D**) CHIKV adhered to C6/36 membrane projections (arrow and arrowhead). Some nucleocapsids appeared to be surrounded by a unique envelope (arrowhead); (n): nucleus; (m): mitochondria; (er): endoplasmic reticulum.

When CHIKV was allowed to infect C6/36 cells at 28 °C, the characteristic features of the infection were apparent. Condensed chromatin (Figure 5A) and extensive vesiculation (Figure 5B) were noted. Virions were found adhering to the cell surface (Figure 5C), and membrane projections contained viral particles that appeared to be clustered within a single envelope bilayer (Figure 5D).

**Figure 6:**
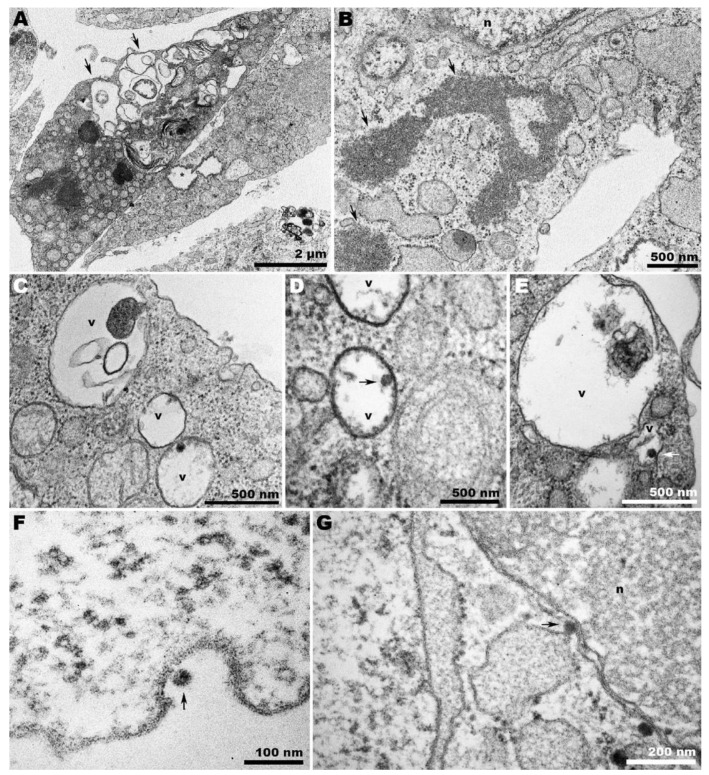
**Transmission electron microscopy of CHIKV-infected C6/36 cells at 37 °C.** (**A**) Panoramic view of infected cells displaying whorled contents within large vacuoles (arrows), vacuoles delimited by loose membranes (asterisk), and (**B**) granular content in the cytoplasm (arrows); (**C**–**E**) viruses (arrows) were scarce within the vacuoles and adhered to the cell surface (**F**). (**G**) CHIKV (arrow) in the perinuclear area; (n): nucleus; (v): vacuole.

At 37 °C, CHIKV-infected C6/36 cells displayed whorled contents within large vacuoles. Vacuoles delimited by loose membranes were also observed (Figure 6A), and granular content was not uncommon in the cytoplasm of these cells (Figure 6B). Despite the extensive vacuolization, only a small number of these vacuoles contained viruses (Figure 6C–E). Regarding C6/36 infected cells at 28 °C, the sporadic intercellular bridges observed were devoid of virions at 37 °C, and few virions were found adhered to the cell surface (Figure 6F). CHIKV was also observed in the perinuclear area (Figure 6G).

**Figure 7:**
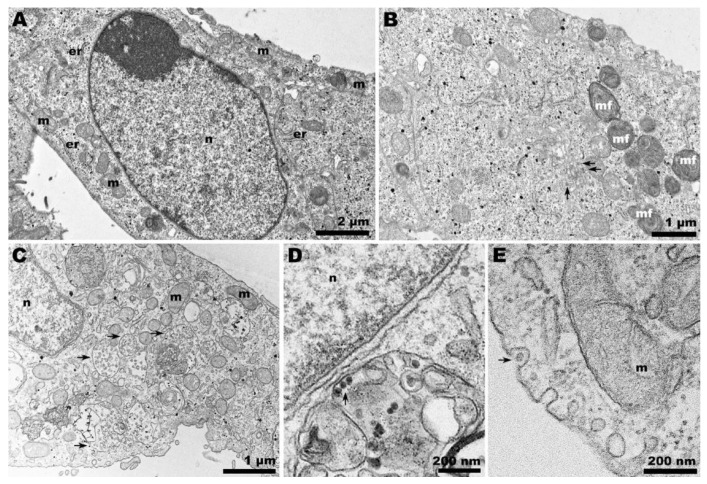
**Transmission electron microscopy of ZIKV-infected Vero cells at 37 and 28 °C.** (**A**,**B**) Panoramic view of Vero cell infected with ZIKV at 37 °C. (**A**) The cell nucleus presented condensed chromatin, and in the ultrathin sections, the virus factories were not clearly noticeable. (**B**) Narrow compartments enclosing viruses (arrows) were observed. (**C**) Panoramic view of Vero cell infected with ZIKV at 28 °C. A profusion of large vacuoles containing vesicles (arrows) was noted; additionally, at 28 °C, viral particles (arrow) were observed adjacent to the nucleus (**D**) and exocytosis-like events (arrow) in the cell periphery (**E**); (n): nucleus; (m): mitochondria; (er): endoplasmic reticulum.

Ultrathin sections of ZIKV-infected Vero cells at 37 °C showed nuclei with condensed chromatin (Figure 7A) and discrete sites of viral factories (Figure 7B). At lower temperatures (28 °C), large vesicle-containing vacuoles were detected (Figure 7C). The presence of ZIKV next to the nucleus was also noted (Figure 7D), in addition to exocytosis-like events (Figure 7E).

**Figure 8:**
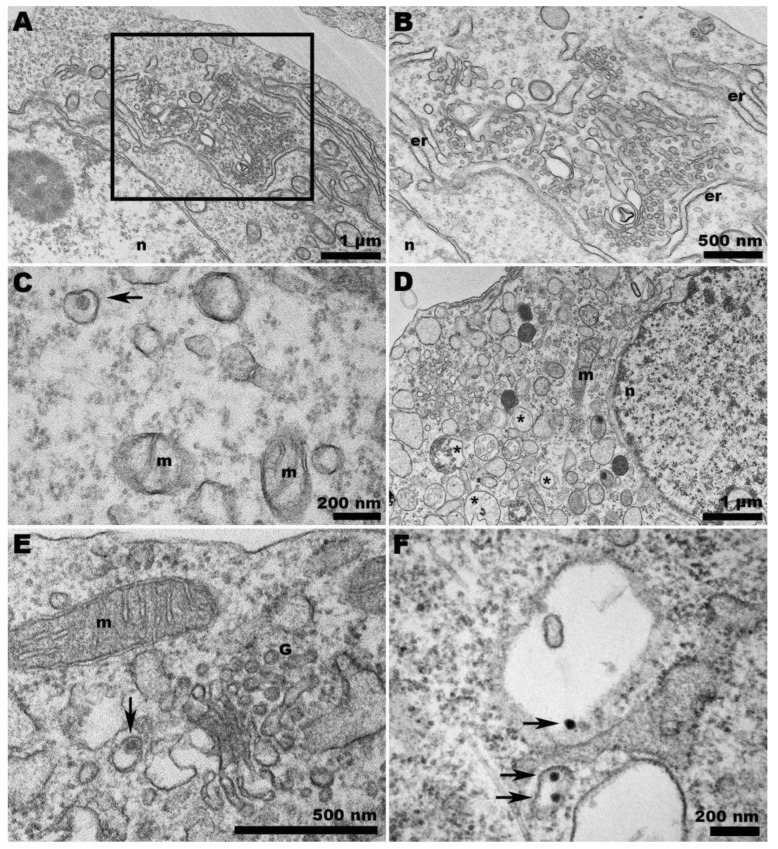
**Transmission electron microscopy of ZIKV-infected C6/36 cells at 28 and 37 °C.** (**A**) Intense membrane rearrangement in the cytosol of ZIKV-infected C6/36 cells at 28 °C. The abundance of spherules is depicted in (**B**). (**C**) ZIKV confined in a vesicle (arrow) in C6/36 cells at 28 °C. (**D**) At 37 °C, ZIKV-infected C6/36 cells exhibited intense vacuolization (asterisks). (**E**) Viruses (arrow) were observed at the Golgi apparatus region but predominantly within the vacuoles (arrows) (**F**); (n): nucleus; (m): mitochondria; (er): endoplasmic reticulum; (G) Golgi apparatus.

When C6/36 cells were infected with ZIKV at the same temperature, we observed the expected profusion of characteristic spherules induced by ZIKV infection (Figure 8A,B). However, the presence of virions within the host cells (Figure 8C) was not as numerous as in the infection in Vero cells.

C6/36 cells infected with ZIKV and incubated at 37 °C displayed a remarkable vacuolization process (Figure 8D). Although the presence of ZIKV was detected in the Golgi complex (Figure 8E), the scarcity of viruses was predominantly observed within the vacuoles (Figure 8F).

## 4. Discussion

Studies on the vector competence for *A. aegypti* and *A. albopictus* from Brazil (and other American countries) have shown a higher infection rate for *A. aegypti* [20]. However, many neglected tropical diseases are caused by arboviruses transmitted by *A. albopictus*, which also exhibits endophilic activity [21]. The higher altitudes reached by these insects may reflect climate change.

Although previous studies have shown, for example, that Dengue virus (DENV) transmission by mosquitoes is reduced due to temperature variations, further studies are necessary to address these effects in the interaction with other arboviruses [15]. It is known that the limiting temperatures for *A. aegypti* are 10–40 °C [21,22], but analogous data are still scarce for *A. albopictus*, except for the fact that this mosquito seems to be more capable of rapidly adapting to new temperatures and consequently spreading to other regions [23]. To contribute to filling this gap, the interaction of *A. albopictus* (C6/36) cells with CHIKV and ZIKV was assessed in the present work.

For CHIKV, higher concentrations of viral RNA and infectious particles were found in C6/36 cells at 28 °C (Figure 1A,B). This is in accordance with previous studies [24] except for 72 hpi, where CHIKV RNA and virions in Vero cells exceeded those found in C6/36.

On the other hand, Vero cells were the most efficient producers of ZIKV, compared to C6/36 cells. It is important to note that, at 24 hpi, while ZIKV RNA is more abundant in the supernatant of C6/36 cells at 28 °C compared to C6/36 cells at 37 °C, this does not reflect the number of infectious particles at the same time point. This can also be noted at 48 hpi, when both temperature conditions are virtually equivalent, yet differ in the number of infectious particles. This distinction is particularly relevant, as many studies tend to interpret RNA assays (PCR) as equivalent to virus titers, which is incorrect. Furthermore, ZIKV production was nearly the same in Vero cells and C6/36 cells at 37 °C, which is consistent with the study conducted by Tesla et al. [25].

While previous works suggest that ZIKV exhibits greater efficacy in infecting or replicating within cells resembling those from its original source [26], our data indicate fluctuations in virus production, depending on the temperature and time of infection (Figure 1C,D).

Positive-stranded RNA viruses traditionally induce the formation of characteristic compartments that harbor virus replication and assembly. CHIKV replication relies on the protrusion of spherules on the plasma membrane surface during the early stages of infection. These spherules are then internalized with the aid of an actin–myosin network and translocated by microtubules to the perinuclear region, where they are added to the surface of acidic compartments, forming the structure known as cytopathic vesicles I (CPV-1) [27].

However, during the late stages of alphavirus infection, the cytoplasm displays a vacuolar system known as cytopathic vesicles II (CPV-II), which originates from the Golgi apparatus and exhibits numerous units of nucleocapsid in its membrane [28]. The presence of these structures indicates ongoing viral morphogenesis, serving as a morphological criterion to verify the success of the infection. We observed CPV-II in Vero cells at 37 °C (Figure 4B and Figure 3A, respectively) but only CPV-I at 28 °C (Figure 4A,B), suggesting a delay in viral morphogenesis.

During CHIKV egress, the occurrence of giant forms on the surface of Vero cells (Figure 3D) contrasted with the eventual envelopment of double nucleocapsids in C6/36 cells (Figure 5D). At 28 °C, infected Vero cells exhibited a distinct pattern of giant forms, with loosened membranes and dispersed capsid-like structures (Figure 4D); however, these were absent in C6/36 at 37 °C.

Since CHIKV exits the cell by budding at the plasma membrane, the presence of already enveloped CHIKV inside peripheral vacuoles is intriguing (Figure 6C,E). However, it is important to note that the scarcity of viruses found within the vacuoles of CHIKV-infected C6/36 cells at 37 °C could be an artifact, as the ultrathin slice may have captured a membrane invagination where the virions lie.

Moreover, although previous studies suggested that in mammalian and insect cells the egress of CHIKV could also occur via exocytosis through CPV-II-derived vesicles containing viruses [29,30], or after intracellular envelopment at specific sites [31], this was not observed even later in the infection (72 hpi). Since our study focused on later periods of infection (>24 h), the spherules were not abundant on the cell surface but were eventually observed due to infection by progeny viruses.

The role of membrane protrusions on CHIKV egress, as previously documented [31], was confirmed herein by the observation of virus budding from these structures in Vero cells (Figure 3D). However, for the first time, this was also shown to occur in C6/36 cells (Figure 5D). It is important to note that although Vero cells infected with CHIKV are capable of projecting intercellular long extensions (ILEs) that promote cell-to-cell viral propagation [32], we could not detect this structure in our observations.

ZIKV morphogenesis involves budding into a modified ER-derived compartment and translocation to the Golgi complex in a secretory pathway toward the extracellular environment [33,34]. Although the canonical ZIKV-induced viral factory [33,35,36] was not clearly apparent in the observed slices of infected Vero cells at 37 °C, the virus was noticed in dilated ER regions (Figure 7A,B).

Differently, a massive vacuolization was observed in infected Vero cells at 28 °C. Vacuolization is also common in ZIKV-infected cells [37], but no ZIKV particles were observed within these vacuoles (Figure 7C). Nevertheless, the virus was present in compartments in the perinuclear regions, and its exocytosis was also observed (Figure 7D,E). This feature is consistent with the infectivity assay we performed (Figure 1D).

As a summary of the concluding remarks, the following points should be addressed:

(i) CHIKV RNA values were higher in C6/36 cells than in Vero cells at both optimal temperatures, at 24 and 48 hpi. However, the values were virtually identical at 72 hpi.

For ZIKV, the number of infectious particles increased linearly in optimal temperatures in both cell types, as expected. However, linearity was also observed at non-optimal temperatures.

These findings suggest better adaptation of C6/36 cells in terms of replication. It is important to note, however, that viral RNA concentrations do not necessarily reflect infectivity. This indicates that during this process, many defective particles may be assembled.

(ii) CHIKV infection in C6/36 cells at 28 °C resulted in a higher quantity of virions at 24 and 48 hpi compared to infection in Vero cells at 37 °C during the same time points.

(iii) For ZIKV, the number of infectious particles increased linearly at optimal temperatures in both cell lines as expected; however, linearity was also observed under non-optimal temperatures.

(iv) The structural arrangement of host cells may influence or reflect the competence for virus production in some way. Vero cells, for instance, exhibited notable vacuolization at 28 °C, a condition in which clustered cells were not observed. Vacuolization was also present in C6/36 cells exposed to 37 °C.

(v) The occurrence of virus surfing and giant forms was abundant in CHIKV-infected Vero cells at 37 °C. CHIKV was also observed in membrane projections of C6/36 cells at 28 °C but not at 37 °C, where few viruses were adhered to the cell surface.

(vi) ZIKV was less prevalent in C6/36 cells compared to Vero cells when both were subjected to optimal temperatures.

## 5. Concluding Remarks

While most studies on the arbovirus cell cycle are conducted under optimal temperature conditions, the influence of global environmental changes can significantly affect the viral cycle within these poikilothermic vectors. Furthermore, it is essential to examine whether the host cell supports the viral cycle at varying temperatures. In this work, we aim to contribute to the understanding of how temperature influences the in vitro cycles of CHIKV and ZIKV in both vertebrate and invertebrate cell types.

## Data Availability

The data that support the findings of this study are available from the corresponding author upon reasonable request.
